# Primary Colorectal Signet-Ring Cell Carcinoma with Synchronous Colonic Metastases in an Asymptomatic Patient: Case Presentation and Comprehensive Literature Review

**DOI:** 10.3390/life16010060

**Published:** 2025-12-30

**Authors:** Oana-Bogdana Barboi, Radu-Alexandru Vulpoi, Diana-Elena Floria, Vadim Rosca, Constantin Simiras, Andriescu Elena-Corina, Amitoaie Iulia, Vasile-Liviu Drug

**Affiliations:** 1 Institute of Gastroenterology and Hepatology, Saint Spiridon Emergency County Hospital, “Grigore T. Popa” University of Medicine and Pharmacy, 700115 Iasi, Romania; oanabarboi@gmail.com (O.-B.B.); iovdiana95@gmail.com (D.-E.F.); vroshca94@gmail.com (V.R.); c_simiras@yahoo.com (C.S.); vasidrug@email.com (V.-L.D.); 2 Histopathology Department, Saint Spiridon Emergency County Hospital, 700111 Iasi, Romania; andriescu_corina@yahoo.co.uk; 3 1st Internal Medicine Clinic, Saint Spiridon Emergency County Hospital, 700111 Iasi, Romania

**Keywords:** colorectal signet-ring cell carcinomas, colonic metastases

## Abstract

Background: Less than 1% of all colorectal cancers (CRCs) are primary colorectal signet-ring cell carcinomas (SRCCs), which represent an uncommon and aggressive histological subtype. Given their subtle onset and rapid progression, these are often diagnosed in an advanced stage, and can be distinguished by the presence of mucin-producing signet-ring cells. Synchronous colonic metastases at initial diagnosis are rather uncommon. Case presentation: We report the case of a 65-year-old male patient who underwent a routine colonoscopy following a positive fecal immunochemical test (FIT). The patient had no remarkable medical history and was asymptomatic. A 3 cm semi-pedunculated polyp and several smaller depressed lesions, 2 cm maximum in diameter, were observed in the descending colon during the colonoscopy. Multiple biopsies were obtained. The lesions were found to be SRCC according to histopathological examination. There was no sign of extra-colonic metastases on the computed tomography (CT). The patient was referred for extensive hemicolectomy, regional lymphadenectomy, and adjuvant chemotherapy. Conclusions: This article provides a thorough literature review on this uncommon presentation and discussion regarding the current understanding of the pathogenesis, clinical manifestations, and management strategies of SRCC. This case highlights the importance of routine screening in detecting aggressive malignancies like SRCC in asymptomatic individuals. Early identification through colonoscopy can lead to timely intervention, potentially improving prognosis.

## 1. Introduction

Colorectal cancer (CRC) poses a significant global health problem, with continuously increasing incidence and prevalence rates. Current estimates indicate that it may become the most frequently diagnosed cancer worldwide within the upcoming years [1]. Several risk factors—including advanced age, genetic factors, dietary patterns, and sedentary lifestyle—contribute to its development [1,2].

Signet-ring cell carcinoma (SRCC) is commonly diagnosed in the stomach, but in 0.5–2% of cases it can also be found in the colon, especially the right segment [3]. First described by Laufman and Saphir in 1951, SRCC remains a rare clinical entity, predominantly among Asian populations [2]. It is found more frequently in young men, with a rather poor prognosis and more aggressive disease course [3]. Regional lymph node invasion and distant metastases occur early, with a 5-year survival rate reaching a maximum of 20% [4].

Metastatic lesions of the colon are exceptionally rare, with very low incidence. These are often incidental findings during colonoscopy and require histopathological confirmation, including specific immunohistochemical staining [5].

## 2. Case Presentation

We report the case of a 65-year-old asymptomatic male patient, with no notable past medical history and no family history of CRC or inflammatory bowel disease. He was referred for further evaluation following a positive routine fecal immunochemical test (FIT). His physical examination was unremarkable. Laboratory investigations—including complete blood count, liver and renal function tests, and inflammatory markers—were within normal ranges, except for a borderline normocytic anemia (hemoglobin 12.5 g/dL).

**Figure 1:**
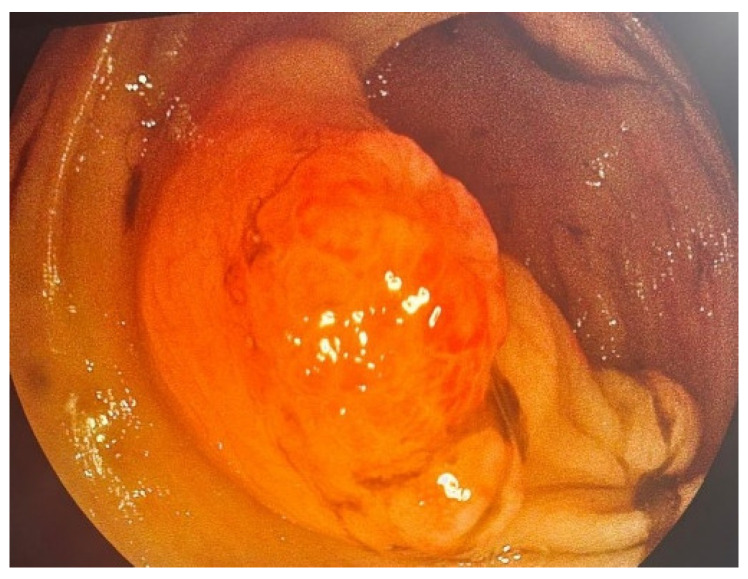
Colonoscopy—semi-pedunculated polyp in the descending colon.

**Figure 2:**
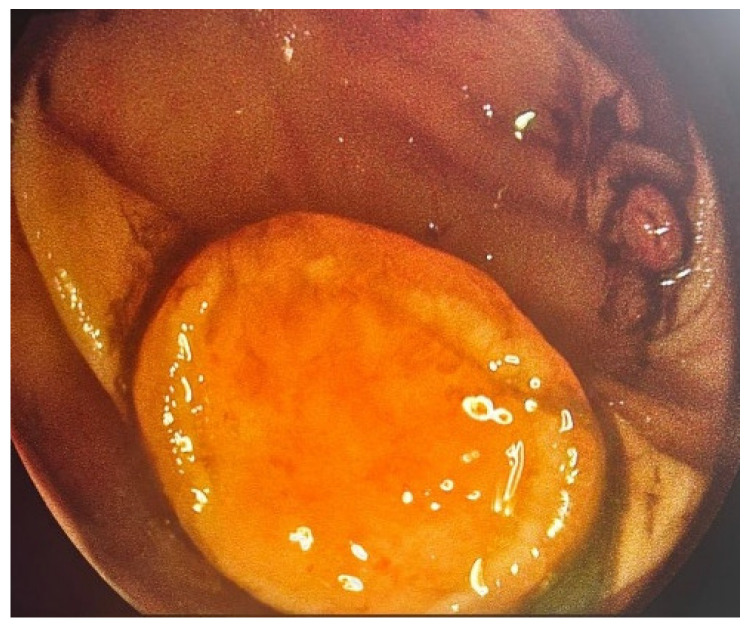
Colonoscopy—depressed lesion in the descending colon.

Colonoscopic evaluation revealed a 3 cm semi-pedunculated polyp (Paris Isp, JNET type 3) (Figure 1) and several smaller depressed lesions, 2 cm maximum in diameter (Paris 0-IIa+c and JNET type 3), in the descending colon. The latter were more than 10 cm from the former lesion. The semi-pedunculated polyp caused partial luminal narrowing without evidence of obstruction (Figure 2). All observed lesions demonstrated endoscopic features suspicious for malignancy. Multiple biopsies were obtained.

**Figure 3:**
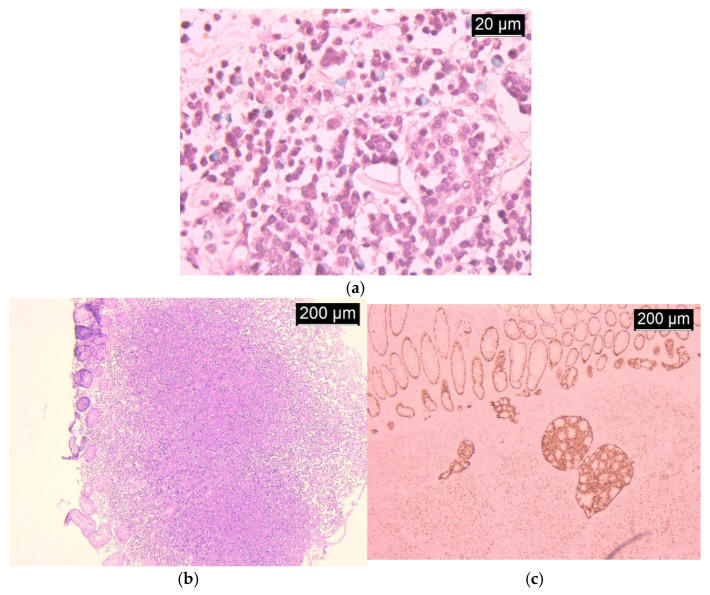
Findings on histopathological examination: (**a**) colonic primary cancer: diffusely infiltrating signet-ring cells embedded within mucinous stroma; (**b**) colonic metastatic tumor: diffusely infiltrating signet-ring cells within mucinous stroma; (**c**) colonic metastatic tumor: immunohistochemical staining positive for CDX2.

Histopathological examination of biopsy specimens highlighted the presence of diffusely infiltrating signet-ring cells embedded within a mucinous stroma. Tumor cells were poorly cohesive, with most exhibiting prominent intracellular mucin vacuoles displacing the nuclei peripherally. Immunohistochemical staining of these specimens showed positivity for CK20 and CDX2, supporting a colorectal origin, and negativity for CK7 and GCDFP-15. Additional immunostaining revealed a preserved expression of E-cadherin, though focal loss was observed in infiltrative areas, suggesting a more aggressive biological behavior. The Ki-67 proliferation index was markedly abnormal at 75%, and p53 showed strong diffuse positivity. Testing for mismatch repair proteins demonstrated intact expression of MLH1, MSH2, MSH6, and PMS2, indicating microsatellite stability (MSS). Further molecular analysis revealed wild-type KRAS and BRAF genes and no evidence of NRAS mutations (Figure 3).

To determine the extent of disease, contrast-enhanced CT examination of the thorax, abdomen, and pelvis was performed. The assessment revealed segmental wall thickening of the descending colon, with locoregional lymphadenopathy, but without peritoneal carcinomatosis or distant metastases. Pelvic magnetic resonance imaging (MRI) was performed to rule out early peritoneal infiltration, considering the characteristic dissemination pattern of this tumor subtype. MRI findings were negative.

The patient was referred for extensive hemicolectomy, regional lymphadenectomy, and adjuvant chemotherapy.

These endoscopic features, alongside the histopathological assessment and immunochemistry, suggested primary colorectal SRCC with synchronous colonic metastases at initial diagnosis in an asymptomatic patient. This finding supports either a skip metastasis mechanism or a possible multifocal origin, although clonal testing was not performed.

## 3. Discussion

Primary SRCC is a rare and aggressive histological subtype of CRC, accounting for 2% of all colorectal malignancies [6,7]. Unlike conventional adenocarcinomas, SRCCs exhibit distinctive cytological features, with tumor cells containing large mucin-filled vacuoles that displace the nucleus peripherally, imparting a signet-ring appearance. This unique histological feature is associated with a more invasive growth pattern and a generally poorer prognosis [8].

SRCC is a rare entity, and published data is scarce, especially for metastatic disease [3]. A study that assessed the incidence of colorectal SRCCs based on patients’ data in the Korea Central Cancer Registry showed the age-standardized incidence rates of colon and rectum SRCC in 2017 were 0.17 and 0.07 per 100,000 individuals, respectively [9]. Kazi et al. evaluated the spatial epidemiology of SRCC to determine regional variations in the proportions of CRCs across India [10]. More than 4000 CRC patients referred to an apex tertiary cancer center in India were mapped based on the geographical location of their residence. The proportion of SRCC among CRC cases varied from 10% in the Northeast region to 19% in the Central and Eastern parts of India. However, despite these statistically significant differences across regions, these findings should be interpreted with caution, given the relatively moderate sample size of the study.

In this report, we present a rare case of primary colorectal SRCC with synchronous colonic metastases in an asymptomatic patient. The presence of synchronous metastases in this patient is consistent with the aggressive disease course of SRCC and underscores the importance of early and comprehensive evaluation, even in the absence of typical gastrointestinal symptoms. Unlike conventional adenocarcinomas, which frequently metastasize to the peritoneum (69%) and liver (23%), regional and distant lymph node metastases are common in SRCC (46.5%). This lymphatic dissemination contributes to advanced stage at diagnosis and poorer outcomes [11].

However, in rare cases like this, metastases can remain confined to the colon, mimicking synchronous primary tumors. The presence of multiple lesions and their identical histopathological features favor an interpretation of intra-colonic metastatic spread, although this phenomenon remains poorly understood. The biological behavior of SRCC is aggressive and unpredictable. A high proliferative index (Ki-67), intact mismatch repair proteins, and wild-type KRAS/BRAF status may suggest a particular molecular phenotype that does not benefit from certain targeted therapies, although more data is needed. Importantly, microsatellite instability, which confers a better prognosis in conventional CRC, is less frequently observed in SRCC [12]. A subset of metastatic SRCC tumors are deficient in mismatch repair (dMMR) (also associated with high microsatellite instability, MSI-H), and some studies report numbers in that general range. SRCC of the colorectum has been reported to have a higher frequency of dMMR/MSI-H than conventional colorectal adenocarcinoma in some studies, though the exact percentage varies by cohort and stage [12].

The synchronous occurrence of primary SRCC and metastatic lesions further complicates clinical management [12,13]. The mechanism behind synchronous metastasis in SRCC remains unclear, although it is hypothesized that these tumors’ high mucin production facilitates early dissemination of cancer cells to distant sites. This highlights the need for advanced imaging and thorough staging procedures at the time of diagnosis to guide treatment planning [14].

Colorectal SRCC is recognized for its poor responsiveness to standard chemotherapy regimens, which tend to be more effective in conventional adenocarcinomas (Objective Response Rate (ORR) in first line 13%, Disease Control Rate (DCR) 50%, 44% with anti-vascular endothelial growth factor (anti-VEGF) and 63% with anti-epidermal growth factor receptor (antiEGFr), Progression-Free Survival (PFS) 5–6 months approximately). [3,15]. This characteristic, coupled with the high metastatic potential of SRCC, requires novel therapeutic strategies. Recent research has explored the use of targeted therapies and immunotherapies, which show promise in improving survival outcomes for patients with SRCC [16,17,18]. A large study including 244,794 patients [19] concluded that signet-ring cell adenocarcinomas of the colon and rectum and mucinous adenocarcinomas of the rectum are associated with poorer survival. The large study of Hugen et al. [20] concluded that the prognostic impact of SRCC is dismal in both colon and rectal cancer patients, but adjuvant chemotherapy is associated with improved survival in adenocarcinoma and SRCC patients. These aggressive histologic variants of colorectal adenocarcinoma should be targeted for research initiatives to improve outcomes. However, more extensive clinical trials are warranted to better understand the role of these therapies in the treatment of SRCC.

The diagnosis of SRCC in an asymptomatic patient raises important questions about the role of screening and early detection. While colorectal cancer screening is standard practice for asymptomatic individuals over the age of 50, the atypical presentation of SRCC complicates detection through conventional screening methods, such as colonoscopy. This case emphasizes the importance of considering rare and atypical malignancies in the differential diagnosis when assessing colorectal tumors, particularly in patients with unusual histological features or unexplained metastatic involvement.

## 4. Conclusions

This case report highlights the rarity and complexity of primary SRCC with synchronous metastases on the left colon. It draws attention to the need for vigilance in diagnosing and managing colorectal cancers with unusual histological features, as well as the importance of a multidisciplinary approach. Further research into more effective treatment approaches for these challenging malignancies is warranted.

## Data Availability

All data supporting the findings of this study are available from the corresponding author upon reasonable request.
